# External validation and update of the International Medical Prevention Registry on Venous Thromboembolism bleeding risk score for predicting bleeding in acutely ill hospitalized medical patients: a retrospective single-center cohort study in Japan

**DOI:** 10.1186/s12959-024-00603-w

**Published:** 2024-03-28

**Authors:** Daichi Arakaki, Mitsunaga Iwata, Teruhiko Terasawa

**Affiliations:** 1 Department of Emergency Medicine and General Internal Medicine, Fujita Health University School of Medicine, 1-98 Dengakugakubo, Kutsukakecho, Toyoake, Achi, 470-1192 Toyoake, Aichi Japan; 2 Department of Emergency and Critical Care, Nagoya University Hospital, Nagoya, Aichi Japan

**Keywords:** Bleeding, IMPROVE, Prediction, Risk assessments models

## Abstract

**Background:**

The International Medical Prevention Registry for Venous Thromboembolism (IMPROVE) Bleeding Risk Score is the recommended risk assessment model (RAM) for predicting bleeding risk in acutely ill medical inpatients in Western countries. However, few studies have assessed its predictive performance in local Asian settings.

**Methods:**

We retrospectively identified acutely ill adolescents and adults (aged ≥ 15 years) who were admitted to our general internal medicine department between July 5, 2016 and July 5, 2021, and extracted data from their electronic medical records. The outcome of interest was the cumulative incidence of major and nonmajor but clinically relevant bleeding 14 days after admission. For the two-risk-group model, we estimated sensitivity, specificity, and positive and negative predictive values (PPV and NPV, respectively). For the 11-risk-group model, we estimated C statistic, expected and observed event ratio (E/O), calibration-in-the-large (CITL), and calibration slope. In addition, we recalibrated the intercept using local data to update the RAM.

**Results:**

Among the 3,876 included patients, 998 (26%) were aged ≥ 85 years, while 656 (17%) were hospitalized in the intensive care unit. The median length of hospital stay was 14 days. Clinically relevant bleeding occurred in 58 patients (1.5%), 49 (1.3%) of whom experienced major bleeding. Sensitivity, specificity, NPV, and PPV were 26.1% (95% confidence interval [CI]: 15.8–40.0%), 84.8% (83.6–85.9%), 98.7% (98.2–99.0%), and 2.5% (1.5–4.3%) for any bleeding and 30.9% (95% CI: 18.8–46.3%), 84.9% (83.7–86.0%), 99.0% (98.5–99.3%), and 2.5% (1.5–4.3%) for major bleeding, respectively. The C statistic, E/O, CITL, and calibration slope were 0.64 (95% CI: 0.58–0.71), 1.69 (1.45–2.05), − 0.55 (− 0.81 to − 0.29), and 0.58 (0.29–0.87) for any bleeding and 0.67 (95% CI: 0.60–0.74), 0.76 (0.61–0.87), 0.29 (0.00–0.58), and 0.42 (0.19–0.64) for major bleeding, respectively. Updating the model substantially corrected the poor calibration observed.

**Conclusions:**

In our Japanese cohort, the IMPROVE bleeding RAM retained the reported moderate discriminative performance. Model recalibration substantially improved the poor calibration obtained using the original RAM. Before its introduction into clinical practice, the updated RAM needs further validation studies and an optimized threshold.

**Supplementary Information:**

The online version contains supplementary material available at 10.1186/s12959-024-00603-w.

## Background

Venous thromboembolism (VTE) is a potentially fatal condition that develops in up to 1% of patients hospitalized for a medical illness (i.e., medical inpatients) in Asian countries [1]. Since pharmacologic thromboprophylaxis holds the risk of critical bleeding [2], several primary studies [3, 4] and systematic reviews [5, 6] have explored prognostic factors and/or risk assessment models incorporating these factors to identify individuals who potentially develop a bleeding event in this context.

Currently, the International Medical Prevention Registry on Venous Thromboembolism (IMPROVE) bleeding risk assessment model (RAM) is the only available externally validated model for predicting bleeding risk in acutely ill medical inpatients [3]. The IMPROVE bleeding RAM, which had been developed using a multivariate model based on an international prospective cohort study involving 12 countries, constitutes a total of 11 items based on nine patient- and disease-related predictors assessed at admission. This RAM predicts the occurrence of major and nonmajor but clinically relevant bleeding 14 days after admission. The linear predictor used in the originally developed mathematical model was simplified into a total score comprising 1–4.5 points per specific item, with each item being stratified into two categorical groups (e.g., presence vs. absence) or three ordinal groups (e.g., low vs. moderate vs. high). The total score used in the original derivation study was further simplified by dichotomizing patients into low- (< 7 points) and high-risk (≥ 7 points) groups based on a threshold of 7 points with expected bleeding risks of 1.5% and 7.9%, respectively. Since the development of the IMPROVE bleeding RAM, only four studies have validated its predictive performance on acutely ill medical inpatients in local settings—two from the United States [7, 8], one from Europe [9] and one from China [10]. However, considering that the transferability of the RAM depends on various contexts, including case variability, settings, and healthcare systems [11], the performance of the IMPROVE bleeding RAM needs to be assessed in the specific population to whom its formal introduction is planned. Therefore, the current study aimed to externally validate the IMPROVE bleeding RAM in acutely ill medical inpatients hospitalized in the general internal medicine department of our university hospital in Japan.

## Methods

This study followed the recommended framework for the external validation of a prediction model [12] and conformed to the Transparent Reporting of a multivariable prediction model for Individual Prognosis or Diagnosis (TRIPOD) statement [13].

### Data source and participants

The external validation was based on a retrospective observational cohort study conducted at Fujita Health University Hospital, a tertiary-care academic hospital in Japan [14]. We retrospectively identified acutely ill medical inpatients admitted to our general internal medicine department between July 5, 2016 and July 5, 2021, and extracted data from their electronic medical records. The eligibility criteria were patients aged ≥ 15 years and hospitalized for ≥ 3 days, and the exclusion criteria were patients with trauma, those who underwent surgery, pregnant women, those on anticoagulation therapy for any reason at admission, and those hospitalized for VTE or bleeding.

### Predictors

One of the investigators (DA), who was blinded to the outcomes, extracted the baseline patient characteristics used in the IMPROVE bleeding RAM [3], including the registered diagnosis procedure combination codes (i.e., equivalent to hospital admission codes) and individually described clinical information at admission, from the identified patients’ medical records. The baseline data included the presence of active gastric or duodenal ulcer, bleeding history < 3 months prior to admission, thrombocyte count, age, international normalized ratio (INR), glomerular filtration rate (GFR) (calculated using the formula 194 × [serum creatinine]^−1.094^ × age^− 0.287^ for men and 194 × [serum creatinine]^−1.094^ × age^− 0.287^ × 0.739 for women), intensive care unit (ICU) or coronary care unit (CCU) admission, use of central venous catheters (CVCs), presence of rheumatic diseases, presence of active malignancy, and sex.

### Interventions for venous thromboembolism prophylaxis

The aforementioned investigator (DA) also extracted data on pharmacological and nonpharmacological prophylactic interventions for VTE during hospitalization, if any, from the patient’s medical records. Pharmacologic prophylaxis was exclusively based on unfractionated heparin, typically 10,000 units per day. We further determined whether a foot pump, either by itself or in combination with heparin, was used for nonpharmacological prophylaxis.

### Risk groups

We used the simplified, score-based version of the IMPROVE bleeding RAM [3], following existing validation studies [7, 8, 10]. The point system used in the RAM, operational definitions of the presence or absence of specific items, and categorization of continuous predictors are provided in Table 1. We initially classified the patients into 11 groups according to their risk of bleeding based on the total RAM scores following the derivation study [3]. Patients were then categorized into two risk groups: low- (those with scores < 7) and high-risk (those with scores ≥ 7) groups.

**Table 1:** International medical prevention registry on venous thromboembolism bleeding risk assessment model^a^

Risk factors	Points
Moderate renal failure (GFR 30–59 vs. **≥**60 ml/min/m^2^)	1
Male vs. Female	1
Age, 40–84 vs. <40	1.5
Current cancer	2
Rheumatic disease	2
Central venous catheter	2
ICU/CCU	2.5
Severe renal failure (GFR < 30 vs. **≥**60 ml/min/m^2^)	2.5
Hepatic failure (INR > 1.5)	2.5
Age, ≥ 85 vs. <40	3.5
Platelet count < 50 × 10^9^	4
Bleeding in the three months before admission	4
Active gastroduodenal ulcer	4.5

^a^ With a total score ≥ 7 (high-risk group) vs. <7 (low-risk group), the predicted event rates for any bleeding and major bleeding at 14 days derived from the derivation study were 7.9% vs. 1.5% and 4.1% vs. 0.4%, respectively. Abbreviations: CCU, coronary care unit; GFR, glomerular filtration rate; ICU, intensive care unit; INR, international normalized ratio

### Outcomes

The outcome of interest was the cumulative incidence of bleeding 14 days after admission. Given that anonymization during data extraction was impossible, an investigator (DA) independently extracted bleeding event data from data on other patient characteristics, extracted on a different occasion, before formally assigning the RAM scores and risk groups. The extracted sources included electronic medical records, including radiology and endoscopy reports, blood transfusion prescription records, laboratory result databases, diagnosis codes assigned after hospitalization, and death certificates. The same investigator determined whether a bleeding event was either major or nonmajor but clinically relevant in all patients. A major bleeding event was defined as bleeding that contributed to death, clinically overt bleeding associated with a 2-g/dL decrease in hemoglobin level or leading to transfusion of more than two units of packed red blood cells, or bleeding within a critical organ, including intracranial, retroperitoneal, intraocular, adrenal gland, spinal, or pericardial bleeding [3]. A nonmajor but clinically relevant bleeding event was defined as overt gastrointestinal bleeding excluding insignificant hemorrhoidal bleeding, gross/macroscopic hematuria lasting > 24 h, substantial epistaxis that required intervention and was recurrent and/or lasted for ≥ 5 min, extensive hematoma or bruising measuring 5 cm in diameter, intra-articular bleeding documented by aspiration, menorrhagia or metrorrhagia with documented increase in quantity or duration, or other bleeding important enough to be recorded into the medical chart [3].

### Sample size

Only 58 bleeding events had been observed in our cohort, which was much lower than the recommended sample size of 100 events [15].

### Missing data

Missing data on thrombocyte count, GFR (with the shifted-log-transformation), and INR (with the Box–Cox transformation) were imputed using multivariate imputation through chained equations [16]. Overall estimates and their 95% confidence intervals (CIs) were calculated, based on 20 imputed datasets [16], using Rubin’s rules [17].

### Statistical analysis

Continuous variables were presented as medians and interquartile ranges (IQRs), whereas categorical variables were presented as numbers and percentages. The cumulative bleeding incidence was estimated 14 days after admission using the Kaplan–Meier method.

For the two-risk-group model, the sensitivity, specificity, and positive and negative predictive values (PPV and NPV, respectively) and their exact 95% CIs were estimated using standard methods.

For the 11-risk-group model, each group was plotted using the standard empirical and fully parametric binormal receiver operating characteristic (ROC) curves obtained based on the probit model with maximum likelihood estimation [18, 19]. The area under the parametric ROC curve was then estimated as the summary discriminatory accuracy (C statistic). The parametric approach has an advantage over the empirical approach in generating parameters to obtain AUC (for a smooth ROC) and CIs that are almost similar to those obtained with nonparametric approaches [20], which can be directly applied for calculating the overall estimates in the presence of missing data. To examine the overall calibration, we estimated (1) the expected and observed event ratio (E/O), (2) calibration-in-the-large (CITL) obtained as the intercept by refitting the logistic regression on the RAM score as the standalone offset term covariate, and (iii) calibration slope as the coefficient for the RAM score [11]. To visualize the calibration for each risk group stratified according to RAM score, we constructed standard calibration plots based on the observed versus expected (i.e., originally reported cumulative bleeding incidence) estimates using a nonparametric locally estimated scatterplot smoothing curve [11].

Recalibrating the baseline risk is an integral additional step for external validation when the calibration performance is suboptimal [12, 21, 22]. This approach is widely accepted as a better alternative to redeveloping a new model *de novo* [23], requiring a large sample size [24]. To update the original 11-risk-group IMPROVE bleeding RAM, we recalibrated the intercept by fitting the original RAM using logistic regression and then constructing the standard calibration plots [12, 25].

All statistical analyses were performed using Stata SE version 18.0 (Stata Corp, College Station, TX, United States) or R version 4.1.2 (R Foundation for Statistical Computing, Vienna, Austria.). Cluster-robust standard errors were obtained using a clustered sandwich estimator to address multiple inclusions of the same patients. To obtain the overall estimates, the calibration slopes and CITLs were combined in the original scale; O/Es and odds ratios (ORs), and proportions and C statistics were transformed into log and logit scales, respectively [11]. All analyses used two-tailed *P*-values, with the level of significance set at *P* < 0.05.

## Results

### Participants

**Fig. 1:**
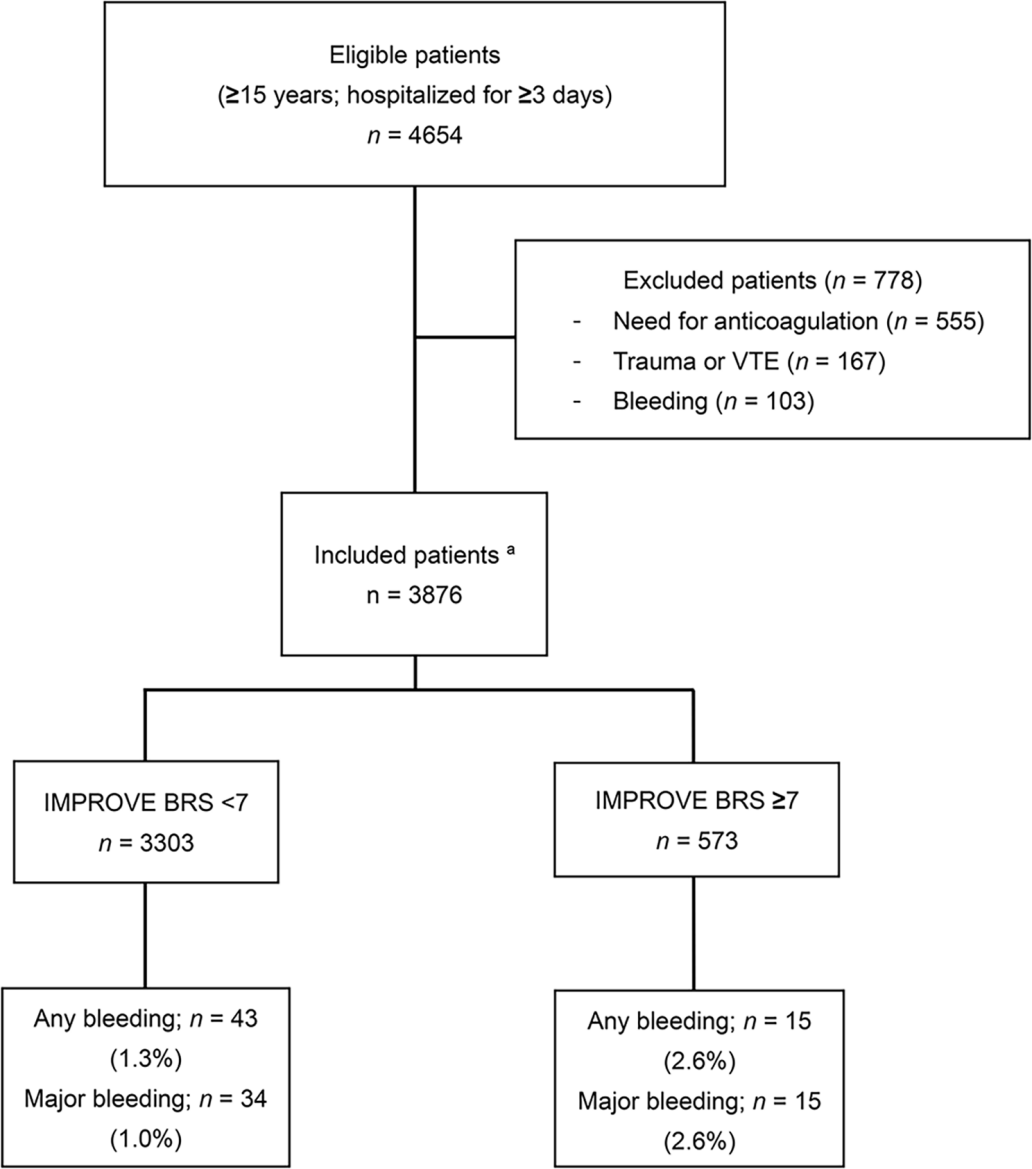
Patient flow diagram. A total of 211, 309, and 230 patients used standalone unfractionated heparin, standalone foot pump, and unfractionated heparin and foot pump for deep vein thrombosis prophylaxis, respectively. BRS, Bleeding Risk Score; IMPROVE, International Medical Prevention Registry on Venous Thromboembolism; VTE, venous thromboembolism

After excluding 778 ineligible patients, our validation cohort comprised a total of 3,876 eligible acutely ill medical inpatients (Fig. 1). Data regarding platelet count, INR, and GFR on admission were missing in 93 (2.4%), 698 (18.0%), and 109 (2.8%) patients, respectively, which were imputed using multiple imputation. Table 2 summarizes the patient characteristics included in the derivation and validation studies of the IMPROVE bleeding RAM along with this cohort. Notably, our cohort had a higher median duration of hospitalization (14 vs. 7 days) and a higher percentage of patients admitted to the ICU or CCU (656/3876 [16.9%] vs. 923/10,866 [8.5%]) and those aged ≥ 85 years (998/3876 [25.7%] vs. 1178/10,866 [10.8%]) than did the derivation cohort. Additionally, our cohort had a lower percentage of patients with active ulcers (3/3876 [0.1%] vs. 236/10,866 [2.2%]), history of recent bleeding events (32/3876 [0.8%] vs. 231/10,866 [2.2%]), and current active cancer (155/3876 [4.0%] vs. 1166/10,866 [10.7%]) than did the derivation cohort. The characteristics of our validation cohort, stratified according to the presence or absence of bleeding events, are presented in Supplemental Table 1.

**Table 2:** Patient characteristics in the derivation and validation studies

Category/Variable	Derivation cohort	Validation cohort				
N (%) or median [IQR]	Decousus et al. [3]	Hostler et al. [8]	Rosenberg et al. [7]	Zhang et al. [10]	Villiger et al. [9]	Present study
Total N	10,866	1,668	12,082	5,076	1,155	3,876
Countries	12 countries ^a^	USA	USA	China	Switzerland	Japan
Duration of hospitalization, days	7 [5–12]	ND	6 [4–10]	11 [8–16]	6.0 [4–10]	14 [8–27]
ICU/CCU admission	923 (8.5)	374 (22.4)	ND (12.5)	340 (6.7)	0 (0)	656 (16.9)
*Demography*						
Age < 40	1,419 (13.1)	234 (14.0)	ND	401 (7.9)	140 (12.1)	301 (7.8)
40–84	8,269 (76.1)	1,144 (68.6)	ND	4,362 (85.9)	902 (78.1)	2,577 (66.5)
≥ 85	1,178 (10.8)	289 (17.3)	ND (11)	313 (6.2)	113 (9.8)	998 (25.7)
Men	5,367 (49.4)	969 (58.1)	ND	2,946 (58.0)	653 (56.5)	2,076 (53.6)
***Medical conditions***						
Active gastroduodenal ulcer	236 (2.2)	34 (2.0)	ND (0.9)	52 (1.0)	22 (1.9)	3 (0.1)
Bleeding 3 months before admission	231 (2.2)	54 (3.2)	ND (0.5)	121 (2.4)	29 (2.5)	32 (0.8)
Platelet count < 50 × 10^9^	179 (1.7)	45 (2.7)	ND	101 (2.0)	21 (1.8)	72 (1.9)
Hepatic failure (INR > 1.5)	219 (2.0)	74 (5.7)	ND (9.2)	115 (2.3)	10 (0.9)	126 (3.3)
GFR < 30 mL/min/m^2^	1,084 (11.0)	218 (13.6)	ND (20.3)	234 (4.6)	101 (8.7)	611 (15.8)
GFR 30–59 mL/min/m^2^	2,520 (25.7)	354 (22.1)	ND	590 (11.6)	298 (25.8)	1,125 (29.0)
GFR ≥ 60 mL/min/m^2^	6,208 (63.3)	1,031 (64.3)	ND	4,252 (83.8)	756 (65.5)	2031 (54.0)
Rheumatic diseases	740 (6.8)	26 (1.6)	ND (15.3)	277 (5.5)	47 (4.1)	71 (1.8)
Current cancer	1,166 (10.7)	361 (21.6)	ND (12.5)	1,196 (23.6)	223 (19.3)	155 (4.0)
Central venous catheter use	820 (7.5)	294 (17.8)	ND (13.8)	319 (6.3)	72 (6.2)	565 (14.6)
***DVT prophylaxis***						
Any interventions	5,686 (52.3)	ND	ND	ND	766 (66.3)	750 (19.3)
Pharmacological intervention alone	5,231 (48.1)	1,235 (74.0)	9,922 (82.1)	432 (8.5) ^g^	745 (64.5) ^d^	211 (5.4)
Nonpharmacological intervention alone	980 (9.0)	652 (39.1) ^d^	ND	ND	62 (5.4) ^d^	309 (8.0)
Bleeding events						
Any bleeding	230 (2.1) ^b^	36 (2.2) ^c^	314 (2.6) ^e^	127(2.5) ^f^	23 (2.0) ^h^	58 (1.5)
Major bleeding	83 (0.8)	23 (1.4) ^c^	232 (1.9) ^e^	38 (0.7) ^f^	8 (0.7) ^h^	49 (1.3)
Nonmajor but clinically relevant bleeding	147 (1.4)	13 (0.8) ^c^	82 (0.7) ^e^	89 (1.8) ^f^	15 (1.3) ^h^	9 (0.2)

a. Australia, Brazil, Canada, Columbia, France, Germany, Italy, Japan, Spain, the United Kingdom, the United States, and Venezuela

b. 3.2% using the Kaplan–Meier method

c. The number of events and rates of any bleeding, major bleeding, and nonmajor but clinically relevant bleeding in the original derivation cohort were 2.7%, 1.9%, and 0.8%, respectively

d. Nonpharmacologic prophylaxis may have been used in conjunction with pharmacological prophylaxis

e. The rates of any bleeding, major bleeding, and nonmajor but clinically relevant bleeding in the original derivation cohort were 2.6%, 1.8%, and 1.6%, respectively

f. The rates of any bleeding, major bleeding, and nonmajor but clinically relevant bleeding in the original derivation cohort were determined to be 2.6%, 1%, and 2%, respectively, using the Kaplan–Meier curve

g. Nonpharmacological prophylaxis may have been used in conjunction with pharmacological prophylaxis

h. Bleeding event within 14 days of admission

CCU, coronary care unit; GFR, glomerular filtration rate; ICU, intensive care unit; IMPROVE, International Medical Prevention Registry on Venous Thromboembolism; INR, international normalized ratio; IQR, interquartile range; ND, no data; RAM, risk assessment model; VTE, venous thromboembolism

### Characteristics of deep vein thrombosis prophylaxis

The derivation cohort received interventions for VTE prophylaxis more frequently in general (5686/10,866 [52.3%] vs. 750/3876 [19.3%]) and pharmacologic interventions in particular (5231/10,866 [48.1%] vs. 211/3876 [5.4%]) than did our cohort (Table 2). Our routine pharmacologic prophylaxis consisted of low-dose intravenous unfractionated heparin (administered at 10,000 units per day) starting on admission and discontinued a few days before discharge.

### Outcomes

**Fig. 2:**
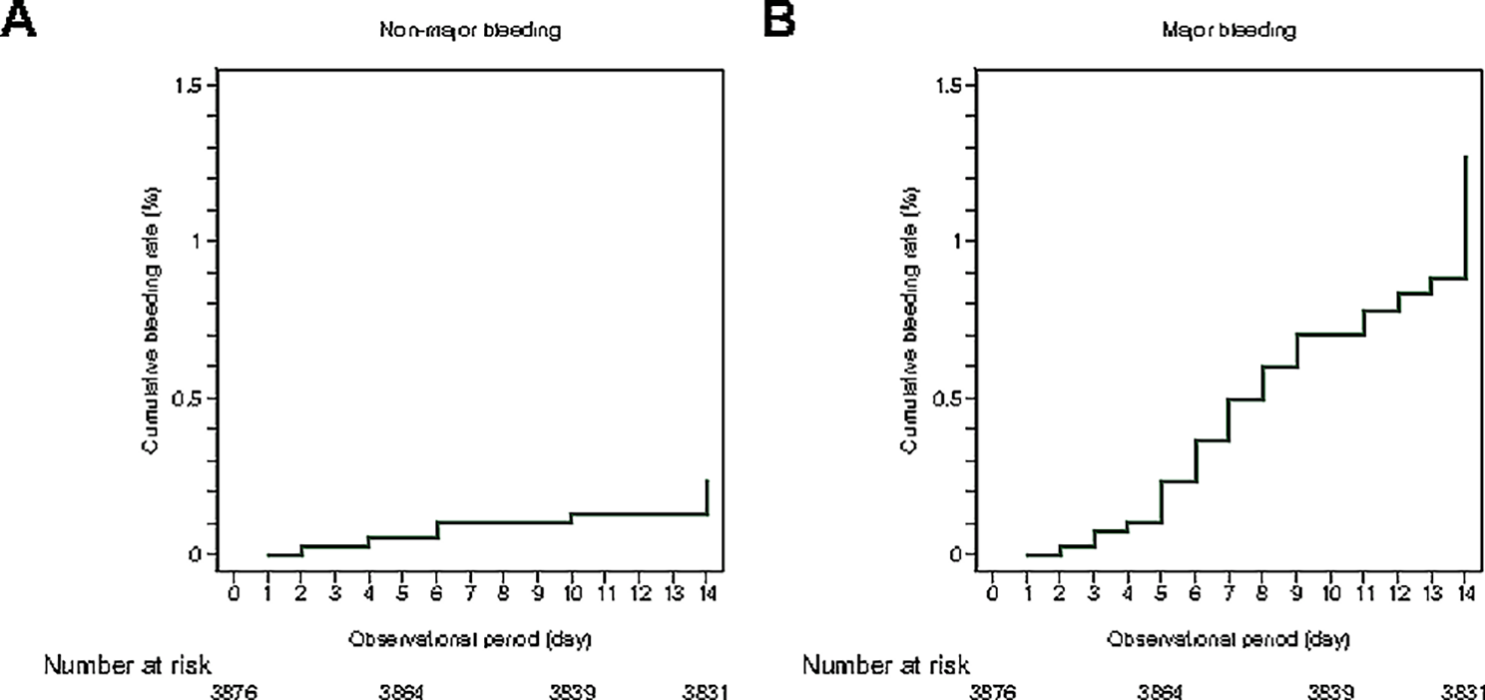
Cumulative incidence of bleeding. Kaplan–Meier plots for nonmajor but clinically relevant bleeding (**A**) and major bleeding (**B**)

Overall, 58 (1.5%) of the 3,876 patients developed any bleeding (49 major and 9 nonmajor but clinically relevant bleeding events) ≤ 14 days after admission (Fig. 2). The 49 major bleeding events occurred in the gastrointestinal tract (38 events); brain (6 events); nongastrointestinal tract intra-abdominal region/organs (2 events); and thorax, nose, and eye (1 event each), whereas the 9 nonmajor but clinically relevant bleeding events occurred in the skin (4 events); uterus (2 events); and urinary tract, eye, and gastrointestinal tract (1 event each). Our cohort had a lower proportion of major bleeding events caused by gastrointestinal ulcers (0/49 [0%] vs. 13/83 [16.0%]) and those occurring in patients with rheumatic diseases (0/49 [0%] vs. 9/83 [10.8%]) or cancer (1/49 [2.0%] vs. 16/83 [19.8%]) than did the derivation cohort (Supplemental Table 2). The incidence of bleeding did not significantly differ between patients who did and did not receive any form of pharmacological prophylaxis (10/441 [2.3%] vs. 48/3435 [1.4%]; *P* = 0.16).

### External validation

A substantial overlap in the distribution of the IMPROVE bleeding scores was observed between patients who did and did not develop bleeding (Supplemental Fig. 1). The median and IQR scores were 5.6 (3.0–9.0) and 4.0 (1.0–11.0) for patients with and without any bleeding and 6.0 (3.0–9.0) and 4.0 (1.0–11.0) for patients with and without major bleeding, respectively.

### Sensitivity, specificity, negative predictive values, and positive predictive values

For the two-risk-group model, in which the low- and high-risk groups had a score < 7 and ≥ 7, respectively, 84.6% (3281/3876) of the entire population was categorized into the low-risk group (Table 3). The cumulative observed event rates for any bleeding were 1.3% (95% CI: 1.0–1.8%) and 2.5% (95% CI: 1.5–4.3%) for the low- and high-risk groups, respectively (OR: 1.98; 95% CI: 1.09–3.58; *P* = 0.025), whereas the cumulative observed event rates for major bleeding were 1.0% (95% CI: 0.7–1.5%) and 2.5% (95% CI: 1.5–4.3%) for low- and high-risk groups, respectively (OR: 2.51; 95% CI: 1.35–4.64; *P* = 0.003). The sensitivity, specificity, NPV, and PPV for any and major bleeding in the two-risk-group model are summarized in Table 4. Although NPV was high, 98.7% (95% CI: 98.2–99.0%) and 99.0% (95% CI: 98.5–99.3%) for any and major bleeding, respectively, PPV was low, 2.5% (95% CI: 1.5–4.3%) and 2.5% (95% CI: 1.5–4.3%) for any and major bleeding, respectively.

**Table 3:** Bleeding rates according to the IMPROVE bleeding RAM^a^

Risk score grouping	Patients	Incidence of any bleeding		Incidence of major bleeding	
	n (%)	n	% (95% CI)	n	% (95% CI)
***Two-risk-group model***					
Low risk (negative)	3,281 (84.6)	43	1.3 (1.0**–**1.8)	34	1.0 (0.7**–**1.5)
High risk (positive)	595 (15.4)	15	2.5 (1.5**–**4.3)	15	2.5 (1.5**–**4.3)
***11-risk group model*** ^b^					
0–1 (0.5)	218 (5.6)	1	0.5 (0.0**–**6.4)	0	0.0 (0.0**–**1.7)
1.5–2 (1.75)	444 (11.5)	1	0.2 (0.0**–**3.3)	1	0.2 (0.0**–**3.3)
2.5 (2.5)	795 (20.5)	7	0.9 (0.4**–**2.1)	5	0.7 (0.3**–**1.8)
3–4 (3.5)	673 (17.4)	12	1.7 (0.9**–**3.2)	10	1.5 (0.7**–**2.9)
4.5–5 (4.75)	656 (16.9)	7	1.1 (0.5**–**2.5)	6	0.9 (0.4**–**2.3)
5.5–6.5 (6)	494 (12.7)	14	2.9 (1.7**–**5.0)	11	2.3 (1.2**–**4.3)
7 (7)	200 (5.2)	3	1.5 (0.4**–**5.5)	3	1.5 (0.4**–**5.5)
7.5–8 (7.75)	151 (3.9)	5	3.4 (1.3**–**8.7)	5	3.4 (1.3**–**8.7)
8.5–9.5 (9)	157 (4.1)	4	2.8 (0.9**–**7.9)	4	2.8 (0.9**–**7.9)
10–12 (11)	58 (1.5)	2	2.7 (0.3**–**20.6)	2	2.7 (0.3**–**20.6)
≥ 12.5	28 (0.7)	1	3.5 (0.2**–**36.5)	1	3.5 (0.2**–**36.5)
Total	3,876 (100)	58	**—**	49	**—**

a. Overall estimates and their 95% CIs based on 20 imputed datasets for missing data synthesized using Rubin’s rules

b. Score intervals (midpoints) defined using the original RAM are presented

CI, confidence interval; IMPROVE, International Medical Prevention Registry on Venous Thromboembolism RAM, risk assessment model

**Table 4:** Sensitivity, specificity, NPV, and PPV using the two-risk-group model

	Predicting any bleeding, % (95% CI)	Predicting major bleeding, % (95% CI)
Sensitivity	26.1 (15.8–40.0)	30.9 (18.8–46.3)
Specificity	84.8 (83.6–85.9)	84.9 (83.7–86.0)
NPV	98.7 (98.2–99.0)	99.0 (98.5–99.3)
PPV	2.5 (1.5–4.3)	2.5 (1.5–4.3)

CI, confidence interval; NPV, negative predictive value; PPV, positive predictive value

### Discrimination performance of the model

**Fig. 3:**
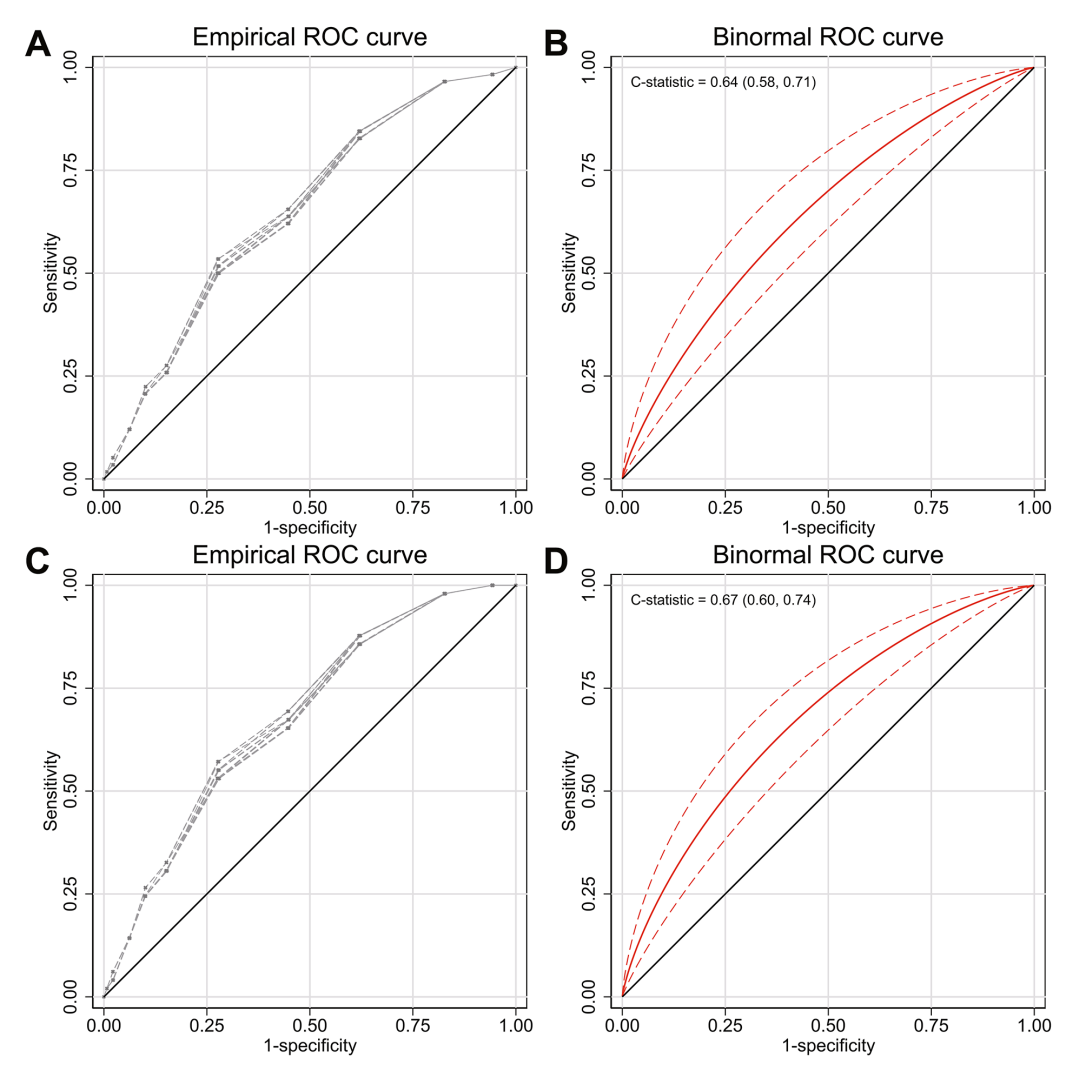
ROC curves for bleeding events. ROC curves are shown for all (**A** and **B**) and major (**C** and **D**) bleeding events. Empirical ROC curves (dashed lines) are based on 20 datasets with imputed missing values. Binomial ROC curves, C statistics, and their respective 95% CIs in parentheses were based on the overall binormal parameters. The ROC curve and 95% CI are depicted by red solid and dashed lines, respectively. CI, confidence interval; ROC, receiver operating characteristic

In the complete, originally reported 11-risk-group model, the cumulative observed event rates for any bleeding ranged from 0.5% for the lowest-risk group to 3.5% for the highest-risk group, whereas the cumulative observed event rates for major bleeding ranged from 0% for the lowest-risk group to 3.5% for the highest-risk group (Table 3). ROC analysis based on the binormal model revealed a C statistic of 0.64 (95% CI: 0.58–0.71) and 0.67 (95% CI: 0.60–0.74) for any and major bleeding, respectively (Fig. 3).

### Calibration performance of the model

**Fig. 4:**
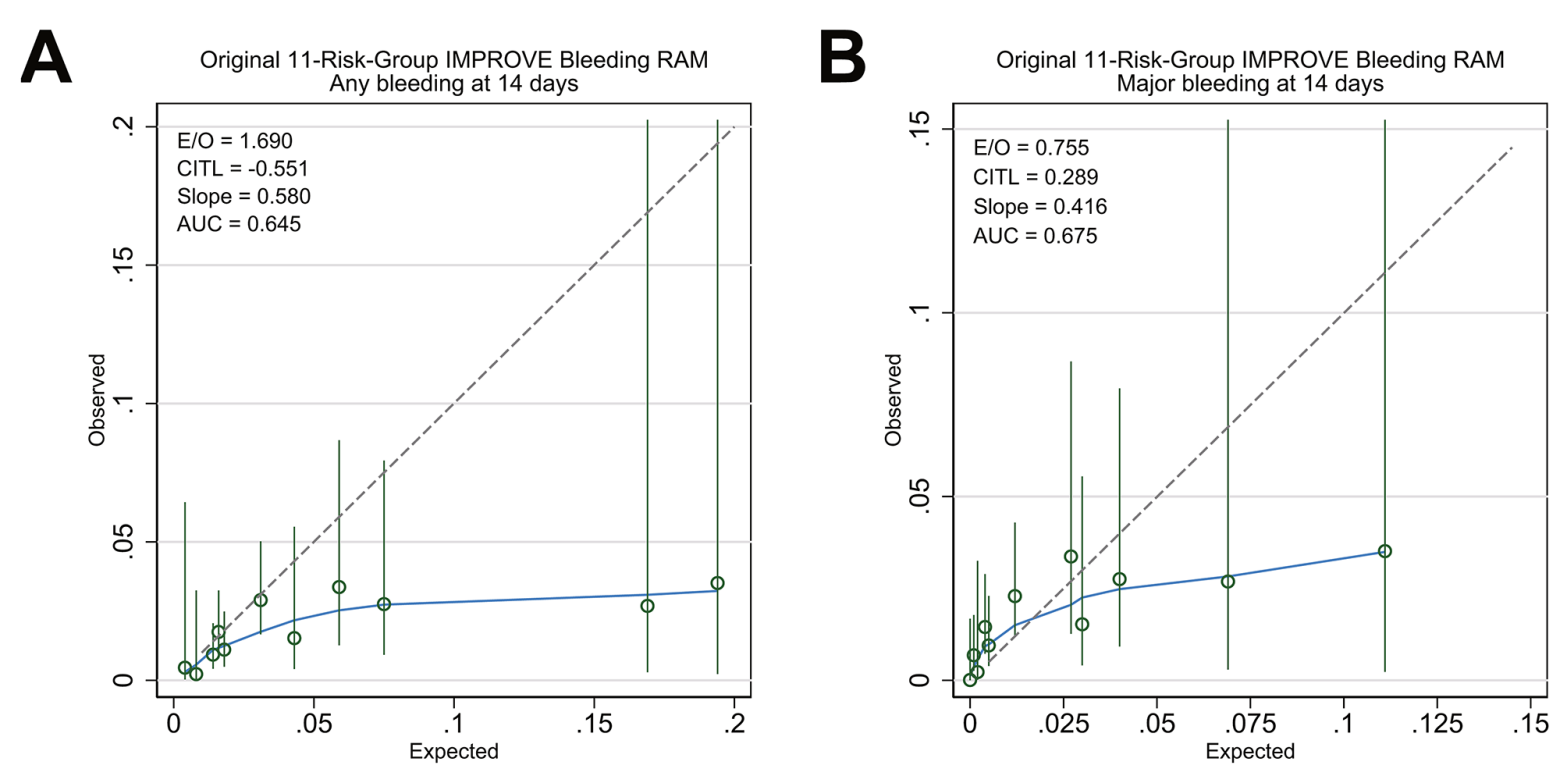
Calibration plots for the original 11-risk group IMPROVE bleeding RAM. Plots for any clinically relevant bleeding (**A**) and major bleeding only (**B**) are shown. Point estimates and their 95% CIs are presented as open green circles and solid vertical lines, respectively. The upper limits of the 95% CI(s) outside the plotted area were truncated. The solid blue line represents the nonparametric locally weighted smoother calibration fit. The gray dashed line represents the reference line for perfect calibration. AUC, area under the (receiver operating characteristic) curve; CI, confidence interval; CITL, calibration-in-the-large; E/O, expected and observed event ratio; IMPROVE, International Medical Prevention Registry on Venous Thromboembolism; RAM, risk assessment model

The overall predicted risks for any bleeding according to the 11-risk-group IMPROVE bleeding RAM were too extreme (calibration slope = 0.58 [95% CI: 0.29–0.87]) and systematically overpredicted (E/O = 1.69 [95% CI: 1.45–2.05]; CITL = − 0.55 (95% CI: −0.81 to − 0.29]). Similarly, the standard calibration plot showed that the observed event rate for each group stratified according to risk was consistently lower than the corresponding predicted event rate (Fig. 4).

The predicted risk for major bleeding was also too extreme (calibration slope = 0.42 [95% CI: 0.19–0.64]); however, the other two calibration measures (CITL and O/E) suggested underprediction (E/O = 0.76 [95% CI: 0.61–0.87]; CITL = 0.29 [95% CI: 0.00–0.58]). In the standard calibration plot, the predicted risks for the lower-risk groups (scores < 7) were underpredicted, whereas those for the higher-risk groups (scores ≥ 7) appeared overpredicted, supporting the overprediction suggested by the overall measures (Fig. 4).

### Model updating

**Fig. 5:**
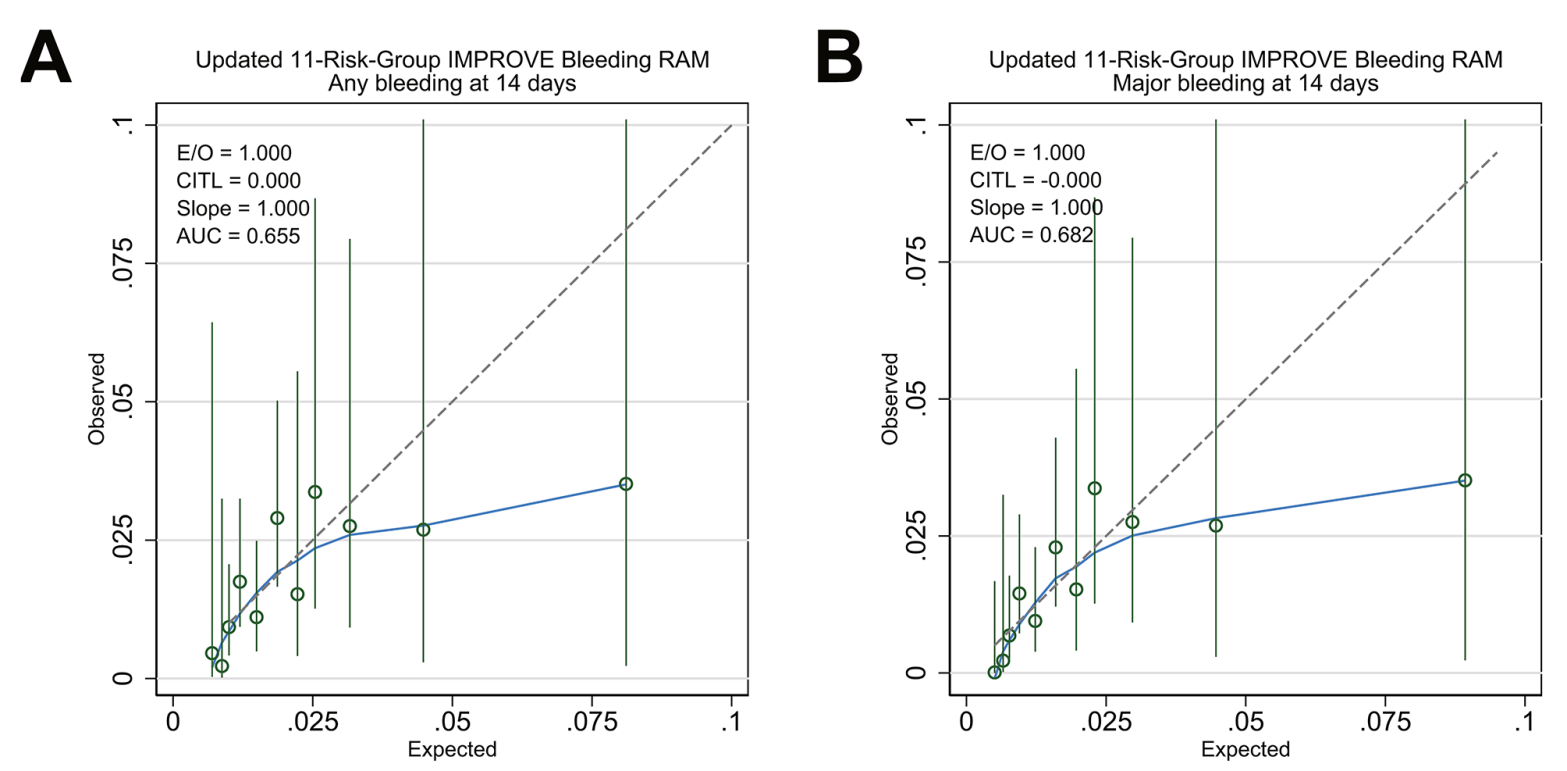
Calibration plots for the updated 11-risk-group IMPROVE bleeding RAM. Plots for any clinically relevant bleeding (**A**) and major bleeding only (**B**) are shown. Point estimates and their 95% CIs are presented as open green circles and solid vertical lines, respectively. The upper limits of the 95% CI(s) outside the plotted area were truncated. The solid blue line represents the nonparametric locally weighted smoother calibration fit. The gray dashed line represents the reference line for perfect calibration AUC, area under the (receiver operating characteristic) curve; CI, confidence interval; CITL, calibration-in-the-large; IMPROVE, International Medical Prevention Registry on Venous Thromboembolism; O/E, observed and expected event ratio; RAM, risk assessment model

Recalibrating the intercept substantially corrected the poor calibration of the original 11-risk-group IMPROVE bleeding RAM. The overall calibration measures suggested that the updated IMPROVE bleeding RAM was well calibrated for predicting any bleeding (E/O = 1.0 [95% CI: 1.0–1.06]; CITL = 0.0 [95% CI: −0.26 to + 0.26]; calibration slope = 1.0 [95% CI: 0.50–1.50]) and major bleeding (E/O = 1.0 [95% CI: 1.00–1.09]; CITL = 0.0 [95% CI: −0.28 to + 0.28]; calibration slope = 1.0 [95% CI: 0.55–1.45]). The expected and observed bleeding rates, as well as standard calibration plots of the updated RAM, also confirmed the corrected calibration for both any and major bleeding (Supplemental Tables 3 and Fig. 5).

## Discussion

This retrospective, single-center cohort study aimed to externally validate the ability of the IMPROVE bleeding RAM to predict an individual’s risk for developing bleeding in acutely ill, medical inpatients admitted to a tertiary-care university hospital in Japan. First, the RAM used in the current study had a moderate but slightly lower discriminative performance than did the RAM used in the derivation study. Second, the RAM was substantially miscalibrated for predicting the risk of bleeding in the present population. Specifically, the RAM systematically overpredicted the risk of any bleeding, underestimated the risk of developing major bleeding in lower-risk patients, and overestimated the risk of developing major bleeding in higher-risk patients. Third, the observed miscalibration of the original RAM was substantially corrected after recalibrating the intercept to update the RAM.

Data from existing validation studies suggest that few differences in model performance are potentially present between Western and Asian populations (Supplemental Tables 4–5) [3, 7–10]. For the full 11-risk group model, existing studies have reported C statistic values ranging from 0.63 to 0.73 for major bleeding events as the measure of discriminative performance, similar to the present study. However, no existing studies have reported formal calibration measures, including E/O, CITL, or calibration slope, which precludes cross-study comparisons. Regarding the two-risk (i.e., high- vs. low-risk) model, the reported rates for any bleeding and major bleeding events in the two risk groups, sensitivity, and specificity varied across studies, suggesting that the optimal threshold of the assigned scores may be context-specific.

### Interpretation

The potential causes of poor calibration include differences in baseline event rates across studies, differences in the effects of included predictors across settings and/or failure to appropriately specify interactions between them, or the possibility that important predictors per se are missing [26]. Updating our model by recalibrating the model intercept (i.e., baseline bleeding risk) substantially improved its calibration performance, leading to an updated E/O of 1 and CITL of 0, coupled with an updated slope of 1. This suggests that the total number of predicted and observed bleeding cases was almost in perfect agreement while maintaining the moderate discriminative performance reported originally, implying that the low baseline bleeding rates in our cohort played (at least) some integral part in the poor calibration [27].

Various factors, such as case variations, distribution of predictors, participants and/or therapeutic interventions potentially affecting the development of bleeding, incidence of bleeding per se, and methods for verifying bleeding events, could affect the model’s predictive performance, consequently increasing or decreasing the predictive performance obtained in the validation setting [28, 29]. Notably, case variation and distribution of predictors in our cohort differed substantially from those in the derivation cohort. Although our patients were older and potentially sicker than the derivation cohort, thus requiring more frequent CVC placements and ICU/CCU admissions and longer hospital stay, fewer had active cancer. However, the prevalence of active ulcers and previous bleeding events within 3 months, two of the three predictors with the largest assigned score weights (i.e., 4–4.5 points), was lower in our patients than in the derivation cohort. Although the distribution of the total RAM scores for the original 11-risk-group and two-risk-group models appeared similar across the two cohorts (Supplemental Tables 4–5), further in-depth assessments were not possible without access to individual-level patient data. Furthermore, the derivation study had a much higher frequency of pharmacologic interventions (approximately 50%) for hospitalized patients than did in the present study (approximately 5%). This difference in clinical background might have also affected the incidence of bleeding events, as well as the predictive performance of the RAM.

### Implications

Empirical studies in other clinical disciplines suggest that RAM performance appears to vary across settings and populations [29, 30]. Therefore, external validation from multiple settings is recommended to comprehensively understand the model’s direct transferability to different contexts and settings [12]. Considering the limited external validation studies, two from Western countries [7, 8] and only one from Asia [10], assessing the reliability and accuracy of the RAM in their local settings, the empirical data from Japan provided by the current study should add to existing data in this field from the perspective of Asian populations. However, the validity of the improved predictive performance of the updated model, particularly in terms of calibration, still needs to be monitored given the changes in the population and measurements over time, which are expected even in the same local setting [27]. Additionally, experts recommend continuous monitoring and dynamic updating of the model performance over time [31].

The applied cutoff threshold of 7 points in the originally reported RAM could have been too low to reasonably differentiate between high-risk and low-risk groups in the present population considering that it achieved a PPV of only 2.5% (vs. a PPV of 4.7–10.9% in the other validation cohorts [7, 8, 10]; Supplemental Table 5). However, the optimal cutoff threshold of RAMs needs to be determined after considering multiple factors, including the risk of bleeding, risk of VTE development, and benefits and harms of pharmacologic VTE prophylaxis therapies. Decision model analysis at this stage could be a candidate approach in this case to best utilize all available resources [32], including the IMPROVE VTE RAM [33], which has been updated specifically for our local setting [14] before formally embarking on impact studies to assess model implementation with a specific criterion to employ pharmacologic VTE prophylaxis in routine clinical practice [34]. For instance, according to the scores assigned using the updated IMPROVE VTE RAM in our local setting demonstrating moderate discriminatory power (C statistic = 0.648), the expected 3-month VTE risks are 3.3% (0 points), 4.8% (1 point), 6.8% (2 points), 9.6% (3 points), 13.5% (4 points), and 18.9% (≥ 5 points) [14].

### Limitations

Our study has several limitations. First, this study was a retrospective analysis based on a single-center experience. Retrospective studies are susceptible to several methodological limitations affecting data reliability, which include nonstandardized, practice-based diagnoses of baseline comorbidities and bleeding outcomes, incorrectly assigned disease codes, and missing data on predictors and outcomes. The extremely low incidence rates of nonmajor but clinically relevant bleeding might have been attributed to our retrospective data extraction (only 0.2% in our study vs. the 0.7–1.8% in the derivation [3] and other validation studies [7, 8, 10]). Furthermore, although our approach in addressing missing data relied on the missing at random assumption, it is still possible that data were not missing at random. Reliable and accurate data extractions can only be achieved with an *a priori* formulated and standardized set of operational definitions.

Second, despite having identified a total of 3,876 patients, our sample size was still small, with only 58 events. A minimum of 100 events is recommended for the external validation of a RAM with a binary outcome [15]. Moreover, the limited number of events had prompted us to recalibrate only the model intercept, which precluded a full model update, including re-estimation of the individual regression coefficients or extension of the model by adding new prognostic factors in addition to recalibrating the model.

Third, similar to previous studies, we failed to consider the effects associated with VTE prophylaxis, such as the use of unfractionated heparin and nonpharmacological interventions. However, this could be reasonably overlooked considering that such effects are typically small [11].

Fourth, validated models even with decent model performance, do not always guarantee to improve individualized healthcare. Prospective validation and impact studies that compare a risk-adapted management strategy with conventional management to assess patient-relevant clinical outcomes are needed to further understand circumstances in which risk-adapted DVT prophylaxis is beneficial. Contextually, the IMPROVE Bleeding RAM should be assessed in light of an individual’s VTE risk, preferably determined using widely validated VTE-specific RAMs.

## Conclusions

In Japanese University cohort of acutely ill medical inpatients, the IMPROVE bleeding RAM retained the moderate discriminative performance that was originally reported and externally validated in the studies from North America, Europe, and China.

### Electronic supplementary material

Below is the link to the electronic supplementary material.

Supplementary Material 1

## Data Availability

The datasets used and/or analyzed during the current study are available from the corresponding author on reasonable request.

## Abbreviations

CCU: coronary care unit
CI: confidence interval
CITL: calibration-in-the-large
CVC: central venous catheter
E/O: expected and observed event ratio
GFR: glomerular filtration rate
ICU: intensive care unit
IMPROVE: International Medical Prevention Registry for Venous Thromboembolism
INR: international normalized ratio
IQR: interquartile range
NPV: negative predictive value
OR: odds ratio
PPV: positive predictive value
RAM: risk assessment model
ROC: receiver operating characteristic
VTE: venous thromboembolism
