# Supplementary material for: External validation and update of the International Medical Prevention Registry on Venous Thromboembolism bleeding risk score for predicting bleeding in acutely ill hospitalized medical patients: a retrospective single-center cohort study in Japan
Source: Thromb J. 2024 Mar 28;22:31. doi: 10.1186/s12959-024-00603-w (PMC10976666; doi:10.1186/s12959-024-00603-w)
Supplement: Supplementary file 1 — Supplementary Material 1 [file 12959_2024_603_MOESM1_ESM.docx]

**Supplementary material**

External validation and update of the IMPROVE bleeding risk score for predicting bleeding in acutely ill hospitalized medical patients: A retrospective single-center cohort study in Japan by Daichi Arakaki, Mitsunaga Iwata, and Teruhiko Terasawa

[Supplementary Figure 1. Score distributions of the IMPROVE bleeding RAM for any clinically relevant bleeding (left panel) and major bleeding only (right panel). 2](#_Toc150613937)

[Supplementary Table 1. Demographic characteristics and distribution of patients by bleeding events. 3](#_Toc150613938)

[Supplementary Table 2. Demographic characteristics and distribution of patients who developed major bleeding events. 4](#_Toc150613939)

[Supplementary Table 3. Bleeding rates by the updated 11-risk IMPROVE bleeding RAM. 5](#_Toc150613940)

[Supplementary Table 4. Published derivation and validation studies of the IMPROBE bleeding RAM (11-risk-group model). 6](#_Toc150613941)

[Supplementary Table 5. Published derivation and validation studies of the IMPROBE bleeding RAM (2-risk-group model). 7](#_Toc150613942)

[Supplementary References 8](#_Toc150613943)

# Supplementary Figure 1. Score distributions of the IMPROVE bleeding RAM for any clinically relevant bleeding (left panel) and major bleeding only (right panel).

IMPROVE = International Medical Prevention Registry on Venous Thromboembolism, RAM = risk assessment model

# Supplementary Table 1. Demographic characteristics and distribution of patients by bleeding events.

| Category/variable, n (%) ^a^ or median value [IQR] | Total | Patients without bleeding | Patients with bleeding | P value ^b^ |
| --- | --- | --- | --- | --- |
| Total N | 3876 | 3818 | 58 |  |
| Duration of hospitalization, days | 14 [8**–**27] | 14 [8**–**27] | 30 [16.5**–**43] | <0.001 |
| ICU/CCU admission | 656 (16.9) | 640 (16.8) | 16 (27.6) | 0.035 |
| ***Demographic data*** |  |  |  |  |
| 40< | 301(7.8) | 300 (7.9) | 1 (1.7) | 0.086 |
| 40–84 | 2577(66.5) | 2534 (66.4) | 43 (74.1) | 0.26 |
| ≥85 | 998(25.7) | 984 (25.8) | 14 (24.1) | 0.88 |
| Men | 2078 (53.6) | 2041 (53.5) | 35 (60.3) | 0.35 |
| ***Medical conditions*** |  |  |  |  |
| Active gastroduodenal ulcer | 3 (0.1) | 3 (0.1) | 0(0) | 1.0 |
| Bleeding <3 months before admission | 32 (0.8) | 31 (0.8) | 1 (1.7) | 0.38 |
| Platelet count <50×10^9^ | 72 (1.9) | 67 (1.8) | 5 (8.6) | 0.001 |
| Hepatic failure (INR>1.5) | 136 (3.5) | 129 (3.4) | 7 (12.1) | 0.001 |
| GFR <30 mL/min/m^2^ | 611 (15.8) | 599 (15.7) | 12 (20.7) | 0.30 |
| GFR 30–59 mL/min/m^2^ | 1143 (29.5) | 1124 (29.4) | 19 (32.8) | 0.58 |
| GFR ≥60 mL/min/m^2^ | 2122 (54.8) | 2095 (54.9) | 27 (46.6) | 0.21 |
| Rheumatic disease | 463(11.9) | 458 (12.0) | 5 (8.6) | 0.54 |
| Current cancer ^c^ | 155 (4.0) | 153 (4.0) | 2 (3.4) | 1 |
| VTE in hospital | 74 (1.9) | 70 (1.8) | 4 (6.9) | 0.024 |
| PE in hospital | 6(0.2) | 6 (0.2) | 0 (0) | 1.0 |
| Central venous catheter use | 565 (14.6) | 549 (14.4) | 16 (27.6) | 0.008 |
| Mortality | 309 (8.0) | 298 (7.8) | 11 (19.0) | 0.005 |
| ***DVT prophylaxis*** |  |  |  |  |
| Any interventions | 750 (19.3) | 734 (19.2) | 16 (27.6) | 0.13 |
| Pharmacological intervention alone | 211 (5.4) | 205 (5.4) | 6 (10.3) | 0.13 |
| Non-pharmacological intervention alone | 309 (8.0) | 303 (7.9) | 6 (10.3) | 0.46 |

1. Data are presented as n (%), unless otherwise indicated.
2. Fisher's exact test or the chi-square test was used to analyze between-group differences.
3. Patients treated with anticancer drugs, with multiple metastases, or receiving palliative care.

CCU = coronary care unit; GFR = glomerular filtration rate; ICU = intensive care unit; IMPROVE = International Medical Prevention Registry on Venous Thromboembolism; INR = international normalized ratio; IQR = interquartile range; ND = no data; RAM = risk assessment model; PE = pulmonary embolism; VTE = venous thromboembolism

# Supplementary Table 2. Demographic characteristics and distribution of patients who developed major bleeding events.

|  | Derivation cohort | Validation cohort | | | | |
| --- | --- | --- | --- | --- | --- | --- |
| Category/Variable (%) ^a^ | Decousus, et al. [1] | Hostler, et al. [2] | Rosenberg, et al. [3] | Zhang, et al. [4] | Villiger, et al. [5] | Present study |
| Patients with major bleeding | 83 | 23 | 232 | 38 | 8 | 49 |
| Age in years |  |  |  |  |  |  |
| 40< | 3 (3.6) | 2 (8.3) | ND | 5 (13.2) | ND | 0 (0) |
| 40-84 | 61 (73.5) | 16 (66.7) | ND | 31 (81.6) | ND | 36 (73.5) |
| ≥85 | 19 (23.0) | 6 (25.0) | ND | 2 (5.3) | ND | 13 (26.5) |
| Men | 40 (48.2) | 8 (33.3) | ND | 22 (57.9) | ND | 31 (63.3) |
| Medical conditions |  |  |  |  |  |  |
| Active gastroduodenal ulcer | 13 (16.0) | 0 (0) | ND | 3 (7.9) | ND | 0 (0) |
| Bleeding <3 months before admission | 6 (7.7) | 5 (20.8) | ND | 9 (23.7) | ND | 1 (2.0) |
| Platelet count <50 × 109 | 9 (11.0) | 2 (8.7) | ND | 8 (21.1) | ND | 5 (8.6) |
| Hepatic failure (INR>1.5) | 8 (9.6) | 4 (17.4) | ND | 5 (13.2) | ND | 7 (12.1) |
| GFR <30 mL/min/m^2^ | 22 (30.0) | 2 (9.1) | ND | 11 (29.0) | ND | 10 (20.4) |
| GFR 30–59 mL/min/m^2^ | 23 (31.0) | 6 (27.3) | ND | 9 (23.7) | ND | 16 (32.3) |
| GFR ≥60 mL/min/m^2^ | 29 (39.0) | 14 (63.6) | ND | 18 (47.4) | ND | 20 (41.0) |
| ICU/CCU admission | 25 (30.1) | 10 (41.7) | ND | 12 (31.6) | ND | 14 (28.6) |
| Central venous catheter | 20 (24.1) | 7 (29.2) | ND | 14 (36.8) | ND | 14 (28.6) |
| Rheumatic disease | 9 (10.8) | 1 (4.2) | ND | 3 (7.9) | ND | 0 (0) |
| Current cancer | 16 (19.8) | 6 (25.0) | ND | 10 (26.3) | ND | 1 (2.0) |

1. Data are presented as n (%), unless otherwise indicated.

CCU = coronary care unit; GFR = glomerular filtration rate; ICU = intensive care unit; IMPROVE = International Medical Prevention Registry on Venous Thromboembolism; INR = international normalized ratio; IQR = interquartile range; ND = no data; RAM = risk assessment model; VTE = venous thromboembolism

# Supplementary Table 3. Bleeding rates by the updated 11-risk IMPROVE bleeding RAM.

| Risk score grouping ^a^ | Patients | Expected risk of any bleeding | Incidence of any bleeding | | Expected risk of major bleeding | Incidence of major bleeding | |
| --- | --- | --- | --- | --- | --- | --- | --- |
|  | *n* (%) | *%* | *n* | *%* (95% CI) | *%* | *n* | *%* (95% CI) |
| 0–1 (0.5) | 218 (5.6) | 0.70 | 1 | 0.5 (0.0**–**6.4) | 0.50 | 0 | 0.0 (0.0**–**1.7) |
| 1.5–2 (1.75) | 444 (11.5) | 0.87 | 1 | 0.2 (0.0**–**3.3) | 0.66 | 1 | 0.2 (0.0**–**3.3) |
| 2.5 (2.5) | 795 (20.5) | 1.00 | 7 | 0.9 (0.4**–**2.1) | 0.77 | 5 | 0.7 (0.3**–**1.8) |
| 3–4 (3.5) | 673 (17.4) | 1.20 | 12 | 1.7 (0.9**–**3.2) | 0.95 | 10 | 1.5 (0.7**–**2.9) |
| 4.5–5 (4.75) | 656 (16.9) | 1.49 | 7 | 1.1 (0.5**–**2.5) | 1.23 | 6 | 0.9 (0.4**–**2.3) |
| 5.5–6.5 (6) | 494 (12.7) | 1.87 | 14 | 2.9 (1.7**–**5.0) | 1.60 | 11 | 2.3 (1.2**–**4.3) |
| 7 (7) | 200 (5.2) | 2.23 | 3 | 1.5 (0.4**–**5.5) | 1.97 | 3 | 1.5 (0.4**–**5.5) |
| 7.5–8 (7.75) | 151 (3.9) | 2.54 | 5 | 3.4 (1.3**–**8.7) | 2.30 | 5 | 3.4 (1.3**–**8.7) |
| 8.5–9.5 (9) | 157 (4.1) | 3.16 | 4 | 2.8 (0.9**–**7.9) | 2.97 | 4 | 2.8 (0.9**–**7.9) |
| 10–12 (11) | 58 (1.5) | 4.48 | 2 | 2.7 (0.3**–**20.6) | 4.47 | 2 | 2.7 (0.3**–**20.6) |
| ≥12.5 | 28 (0.7) | 8.11 | 1 | 3.5 (0.2**–**36.5) | 8.93 | 1 | 3.5 (0.2**–**36.5) |
| Total | 3876 (100) | **—** | 58 | **—** | **—** | 49 | **—** |

1. Score intervals (midpoints) are presented.

CI = confidence interval; IMPROVE = International Medical Prevention Registry on Venous Thromboembolism; RAM = risk assessment model

# Supplementary Table 4. Published derivation and validation studies of the IMPROBE bleeding RAM (11-risk-group model).

| Source [ref] Country | N | Cumulative bleeding rates, n (%) | | Distribution of patients assigned to the lowest- to highest-risk groups [risk score], n (%) | | | | | | | | | | | % range of bleeding rates | | *C*-statistic | | E/O; CITL; slope for bleeding events | |
| --- | --- | --- | --- | --- | --- | --- | --- | --- | --- | --- | --- | --- | --- | --- | --- | --- | --- | --- | --- | --- |
|  |  | Any | Major | 1^st^ [0-1] | 2^nd^ [1.5-2] | 3^rd^ [2.5] | 4^th^ [3-4] | 5^th^ [4.5-5] | 6^th^ [5.5-6.5] | 7^th^ [7] | 8^th^ [7.5-8] | 9^th^ [8.5-9.5] | 10^th^ [10-12] | 11^th^ [>12] | Any | Major | Any | Major | Any | Major |
| Decousus 2011 [1] ^a^  12 countries | 10866 | 230 (3.2) | 83 (1.2) | 778 (8.3) | 1,448 (15.4) | 2,340 (24.9) | 1,539 (16.4) | 1,466 (15.6) | 905 (9.6) | 300 (3.2) | 220 (2.3) | 226 (2.4) | 130 (1.4) | 36 (0.4) | 0.4 - 19.4 | 0 - 11.1 | 0.71 | NR | NA | NA |
| Rosenberg 2016 [3]  USA | 12082 | 314 (2.6) | 232 (1.8) | 352 (2.9) | 1,102 (9.1) | 1,829 (15.2) | 2,062 (17.1) | 2,472 (20.5) | 2,004 (16.6) | 677 (5.6) | 571 (4.7) | 597 (4.9) | 322 (2.7) | 73 (0.6) | 0.3 - 11.0 | 0.3 - 6.8 | 0.63 | NR | NR | NR |
| Hostler 2016  USA [2] | 1668 | 45 (2.7) | 31 (1.9) | NR | NR | NR | NR | NR | NR | NR | NR | NR | NR | NR | NR | NR | NR | NR | NR | NR |
| Zhang 2020 [4]  China | 5076 | 127 (2.6) | 38 (0.7) | 113 (2.2) | 236 (4.6) | 1092 (21.5) | 1565 (30.8) | 708 (13.9) | 850 (16.7) | 84 (1.7) | 208 (4.1) | 120 (2.4) | 81 (1.6) | 19 (0.4) | 0.9 - 26.3 | 0 - 21.1 | 0.73 | NR | NR | NR |
| Villiger 2023 [5]  Switzerland ^c^ | 1155 | 23 (2.0) | 8 (0.7) | 65 (5.6) | 198 (17.1) | 358 (31.0) | 135 (11.7) | 151 (13.1) | 93 (8.1) | 66 (5.7) | 45 (3.9) | 44 (3.8) | **—** | **—** | NR | 1.1 - 24.6 | NR | 0.73 | NR | NR |
| Present study  Japan | 3876 | 58 (1.5) | 49 (1.3) | 218 (5.6) | 444 (11.5) | 795 (20.5) | 673 (17.4) | 656 (16.9) | 494 (12.7) | 200 (5.2) | 151 (3.9) | 157 (4.1) | 58 (1.5) | 28 (0.7) | 0.5 - 3.5^b^ | 0 - 3.5^b^ | 0.65 | 0.68 | 1.69; -0.55; 0.58 | 0.76; 0.29; 0.42 |

1. Data are also extracted from the report by Rosenberg 2016 [3].
2. % range of expected any and major bleeding rates were 0.7-8.1% and 0.5-8.9%, respectively.
3. 8-risk group model based on a differently defined risk-score groups (0-0.5, 1-1.5, 2-2.5, 3-3.5, 4-4.5, 5-5.5, 6-6.5, 7-7.5, and 8 or greater) was used.

Any = any clinically relevant bleeding; CITL calibration-in-the-large; E/O = expected and observed event ratio; NA = not applicable; NPV = negative predictive value; NR = not reported; PPV = positive predictive value

# Supplementary Table 5. Published derivation and validation studies of the IMPROBE bleeding RAM (2-risk-group model).

| Source [ref]  Country | N | Cumulative bleeding rate, n (%) | | Low risk | | | High risk | | | Any bleeding, % | | | | Major bleeding, % | | | |
| --- | --- | --- | --- | --- | --- | --- | --- | --- | --- | --- | --- | --- | --- | --- | --- | --- | --- |
|  |  | Any | Major | n (%) | Bleeding rate, % | | n (%) | Bleeding rate, % | | Se | Sp | PPV | NPV | Se | Sp | PPV | NPV |
|  |  |  |  |  | Any | Major |  | Any | Major |  |  |  |  |  |  |  |  |
| Decousus 2011 [1] ^a^  12 countries | 10866 | 230 (3.2) | 83 (1.2) | 8476 (90.3) | 1.5 | 0.4 | 912 (9.7) | 7.9 | 4.1 | 35.9 | 90.9 | 2.6 | 98.2 | 51 | 90 | 4 | 99.0 |
| Rosenberg 2016 [3]  USA | 12082 | 314 (2.6) | 232 (1.8) | 9821 (81.4) | 2.1 | 1.5 | 2240 (18.6) | 4.7 | 3.2 | 34 | 81.5 | 4.7 | 97.9 | 33.3 | 81.3 | 3.2 | 98.5 |
| Hostler 2016  USA [2] | 1668 | 45 (2.7) | 31 (1.9) | 1301 (78.0) | 2.7 | 1.6 | 367 (22.0) | 6.5 | 5.4 | NA | NA | NA | NA | NA | NA | NA | NA |
| Zhang 2020 [4]  China | 5076 | 127 (2.6) | 38 (0.7) | 4564 (89.9) | 1.6 | 0.3 | 512 (10.1) | 10.9 | 4.5 | 44.1 | 90.8 | 10.9 | 98.4 | 60.5 | 90.3 | 4.5 | 99.0 |
| Villiger 2023 [5]  Switzerland | 1155 | 23 (2.0) | 8 (0.7) | 1066 (92.3) | 1.3 | 0.4 | 89 (7.7) | 9 | 3.3 | 34.8 | 92.8 | 9.0 | 98.6 | 37.5 | 92.5 | 3.4 | 99.5 |
| Present study  Japan | 3876 | 58 (1.5) | 49 (1.3) | 3281 (84.6) | 1.3 | 1.0 | 595 (15.4) | 2.5 | 2.5 | 26.1 | 84.8 | 2.5 | 98.7 | 30.9 | 84.9 | 2.5 | 99.0 |

1. Data are also extracted from the report by Rosenberg 2016 [3].
2. % range of expected any and major bleeding rates were 0.7-8.1% and 0.5-8.9%, respectively.

Any = any clinically relevant bleeding; CITL = calibration-in-the-large; E/O = expected and observed event ratio; Major = major bleeding; NA = not applicable; NPV = negative predictive value; NR = not reported; PPV = positive predictive value; Se = sensitivity; Sp = specificity

# Supplementary References

1. Decousus H, Tapson VF, Bergmann JF, Chong BH, Froehlich JB, Kakkar AK, Merli GJ, Monreal M, Nakamura M, Pavanello R *et al*: **Factors at admission associated with bleeding risk in medical patients: findings from the IMPROVE investigators**. *Chest* 2011, **139**(1):69-79.

2. Hostler DC, Marx ES, Moores LK, Petteys SK, Hostler JM, Mitchell JD, Holley PR, Collen JF, Foster BE, Holley AB: **Validation of the International Medical Prevention Registry on Venous Thromboembolism Bleeding Risk Score**. *Chest* 2016, **149**(2):372-379.

3. Rosenberg DJ, Press A, Fishbein J, Lesser M, McCullagh L, McGinn T, Spyropoulos AC: **External validation of the IMPROVE Bleeding Risk Assessment Model in medical patients**. *Thromb Haemost* 2016, **116**(3):530-536.

4. Zhang Z, Zhai Z, Li W, Qin X, Qu J, Shi Y, Xu R, Xu Y, Wang C, Dissol VEi: **Validation of the IMPROVE bleeding risk score in Chinese medical patients during hospitalization: Findings from the dissolve-2 study**. *Lancet Reg Health West Pac* 2020, **4**:100054.

5. Villiger R, Julliard P, Darbellay Farhoumand P, Choffat D, Tritschler T, Stalder O, Rossel J-B, Aujesky D, Méan M, Baumgartner C: **Prediction of in-hospital bleeding in acutely ill medical patients: External validation of the IMPROVE bleeding risk score**. *Thrombosis Research* 2023, **230**:37-44.
